# Simple Cystatin C Formula for Estimation of Glomerular Filtration Rate in Overweight Patients with Diabetes Mellitus Type 2 and Chronic Kidney Disease

**DOI:** 10.1155/2012/179849

**Published:** 2012-09-12

**Authors:** Sebastjan Bevc, Radovan Hojs, Robert Ekart, Matej Završnik, Maksimiljan Gorenjak, Ludvik Puklavec

**Affiliations:** ^1^Department of Nephrology, Clinic of Internal Medicine, University Medical Center Maribor, 2000 Maribor, Slovenia; ^2^Department of Endocrinology and Diabetology, Clinic of Internal Medicine, University Medical Center Maribor, 2000 Maribor, Slovenia; ^3^Department of Clinical Chemistry, Clinic of Internal Medicine, University Medical Center Maribor, 2000 Maribor, Slovenia; ^4^Department of Nuclear Medicine, Clinic of Internal Medicine, University Medical Center Maribor, 2000 Maribor, Slovenia

## Abstract

In clinical practice the glomerular filtration rate (GFR) is estimated from serum creatinine-based equations like the Cockcroft-Gault formula (C&G) and Modification of Diet in Renal Disease formula (MDRD). Recently, serum cystatin C-based equations, the newer creatinine formula (The Chronic Kidney Disease Epidemiology Collaboration formula (CKD-EPI)), and equation that use both serum creatinine and cystatin C (CKD-EPI creatinine & cystatin formula) were proposed as new GFR markers. Present study compares serum creatinine-based equations, combined (including both serum creatinine and cystatin C) equation, and serum simple cystatin C formula (100/serum cystatin C) against 51CrEDTA clearance in 113 adult overweight Caucasians with diabetes mellitus type 2 (DM2) and chronic kidney disease (CKD). The results of present study demonstrated that the simple cystatin C formula could be a useful tool for the evaluation of renal function in overweight patients with DM2 and impaired kidney function in daily clinical practice in hospital and especially in outpatients. Despite the advantages of the simple cystatin C formula, cystatin C-based equations cannot completely replace the “gold standard” for estimation of the GFR in a population of DM2 patients with CKD, but may contribute to a more accurate selection of patients requiring such invasive and costly procedures.

## 1. Introduction

Chronic kidney disease (CKD) is an important public health problem classified into stages according to the level of GFR. Therefore, estimation of the GFR is essential for the evaluation of patients with CKD and is useful tool to screen for chronic kidney disease also in high-risk groups as persons with diabetes mellitus. GFR estimation allows us to detect early impairment of kidney function, prevent further deterioration and complications, correct the dosage of drugs cleared by the kidney so as to avoid potential drug toxicity, and manage CKD patients. Recently, the National Kidney Disease Education Program (NKDEP) recommended reporting GFR values above 60 mL/min/1.73 m^2^ not as an exact number but simply as >60 mL/min/1.73 m^2^, and contrary for the values of 60 mL/min/1.73 m^2^ and below the exact numerical estimate should be reported [1]. For clinicians the GFR below 60 mL/min/1.73 m^2^ is very important. The values indicate the presence of CKD and represent an increased risk of impaired kidney function, progression to kidney failure, and premature death caused by cardiovascular events of patients with CKD [2, 3].

Over the last decades several different markers for estimation of GFR have been proposed. Despite all known disadvantages, serum creatinine concentration and predictive equations, such as the Cockcroft-Gault (C&G) formula and abbreviated modification of diet in renal disease (MDRD) formula, have become the most commonly used marker to estimate GFR in clinical practice as in most studies [4–6]. Furthermore, estimation of GFR derived from MDRD formula is recommended in annual evaluation of all patients with type 2 diabetes mellitus (DM2) [7]. Unfortunately, both these formulas are also limited by lack of validation in the full range of GFR to which they are applied [8]. To minimize some of these limitations, such as imprecision and systematic underestimation of measured GFR with MDRD formula, new the Chronic Kidney Disease Epidemiology Collaboration equation (CKD-EPI formula) was developed [9]. The authors of the new equation validated CKD-EPI equation using data pooled from several previous studies and showed that the new formula is more accurate than the widely used MDRD formula [9]. Common features of these equations are reliant on serum creatinine and demographic and anthropometric data, and the accuracy of these formulas is still debated [6, 8].

Recently, serum cystatin C low-molecular-weight protease inhibitor, that is freely filtered across the glomerular membrane and then reabsorbed and metabolized in the proximal tubule, was proposed as a new endogenous marker of GFR [10, 11]. The previous reports have suggested that serum cystatin C concentration is a better indicator of GFR than serum creatinine concentration in patients with spine injury, liver cirrhosis, diabetes, mild to moderate impaired kidney function, and in elderly patients [12–16]. As alternatives to serum creatinine-based equations several serum cystatin C-based equations (cystatin C formulas) have been developed and proposed to estimate the GFR [17–24]. Additionally, some authors have also recently advanced the hypothesis that an equation combining filtration markers (serum creatinine and serum cystatin C) may be useful. CKD Epidemiology Collaboration formula is one of proposed new equations that use both serum creatinine and serum cystatin C (CKD-EPI creatinine and cystatin formula) for estimation of kidney function [25, 26].

According to results of some previously published studies body weight may explain variability in performance between the C&G and MDRD formulas in overweight patients with diabetes mellitus type 2 (DM2) [27, 28]. However, less is known about the ability of newer equations, like CKD-EPI formulas and serum cystatin C-based equations, to estimate the GFR in overweight patients with DM2.

The aim of our study was to compare three serum creatinine-based equations (C&G formula, MDRD formula, CKD-EPI formula), CKD-EPI creatinine and cystatin formula and the simple cystatin C formula against ^51^CrEDTA clearance in overweight patients with DM2 and CKD. 

## 2. Patients and Methods

In this study 113 adult Caucasians overweight (BMI > 25 kg/m^2^) patients (43 women and 70 men) with DM2 and CKD were included. All patients were referred for ^51^CrEDTA clearance by nephrologists, diabetologists, cardiologists, or general internists because of suspected or established renal dysfunction. At the same time as ^51^CrEDTA clearance was estimated both serum creatinine and serum cystatin C were measured. Serum creatinine was measured by using the kinetic method according to the Jaffé method without deproteinisation (Roche Diagnostics). This is a compensated method based on manufacturer instructions and was described previously [29]. Serum cystatin C was measured by the particle-enhanced immunonephelometric method (Dade Behring), serum cystatin C assay traceable to the reference material. The GFR was estimated from a single ^51^CrEDTA injection and three blood samples (120, 180, and 240 minutes after parenteral application of the marker) according to committee on renal clearance recommendations [30]. ^51^CrEDTA clearance was calculated in mililitre per minute per 1.73 m^2^. The GFR was calculated according to C&G (i), MDRD (ii), and CKD-EPI (iii) formulas:

 (i) GFR calculated according to C&G formula:

(1) $$\begin{array}{c} \frac{\left[140-\text{age }\left(\text{years}\right)\right]\times \text{ body weight }\left(\text{kg}\right)}{\left[0.815\times \text{serum}\;\text{creatinine }\left(\mu \text{mol}/\text{L}\right)\right]}. \end{array}$$

The correction factor of 0.85 was used for women.

(ii) GFR calculated according to MDRD formula:

(2) $$\begin{array}{c} 175\times \text{serum}\;\text{creatinine}\;{\left(\text{mg}/\text{dL}\;\right)}^{-1.154}\times \text{age}\;{\left(\text{years}\right)}^{-0.203}. \end{array}$$

The correction factor of 0.742 was used for women.

(iii) GFR calculated according to CKD-EPI formula:

(3) $$\begin{array}{c} \text{GFR}=a\times {\left(\frac{\text{serum}\;\text{creatinine}\;\;\left(\text{mg}/\text{dL}\right)}{b}\right)}^{c}\times {\left(0.993\right)}^{\text{age}}. \end{array}$$

The variable *a* takes on the following values on the basis of race and sex: black women = 166, black men = 164, white/other women = 144, white/other men = 141.

The variable *b* takes on the following values on the basis of sex: women = 0.7, men = 0.9.

The variable *c* takes on the following values on the basis of sex and creatinine measurement: women: serum creatinine ≤ 0.7 mg/dL = −0.329, serum creatinine > 0.7 mg/dL = −1.209, men: serum creatinine ≤ 0.7 mg/dL = −0.411, serum creatinine > 0.7 mg/dL = −1.209.

(iv) GFR calculated according to CKD-EPI creatinine and cystatin formula:

(4) 177.6×(serum  creatinine(mgdL))−0.65×(serum  cystatin  C(mgL))−0.57×age−0.2.  

The correction factor of 0.82 was used for women.

The C&G formula was standardized for a 1.73 m^2^ body surface area (according to the DuBois and DuBois method). The MDRD formula and CKD-EPI formulas are already standardized for a 1.73 m^2^ body surface area.

GFR wascalculated also according to previously published simple cystatin C formula (iv) [31].

(v) GFR calculated according to the simple cystatin C formula:

(5) $$\begin{array}{c} \frac{100}{\text{serum}\;\text{cystatin C }\left(\text{mg}/\text{L}\right)}. \end{array}$$

In the statistical analysis SPSS for Windows (version 12.0.1) and MedCalc for Windows (version 5.00.020) were used. The mean values, range, and SD were calculated. Pearson's correlation coefficient was used for defining the correlation between ^51^CrEDTA clearance and serum creatinine, serum cystatin C, the GFR calculated from the serum creatinine-based formulas, the GFR calculated from the cystatin C formula and the GFR calculated from combined formula (including both serum creatinine and cystatin C). In order to determine the diagnostic accuracy of the serum cystatin C-based formula in comparison with the other markers of GFR receiver-operating characteristic (ROC) plots were constructed and analysed. The area under the curve describes the test's overall performance and is used to compare different tests. Sensitivity and specificity were calculated. The GFR determined with ^51^CrEDTA clearance was used as the gold standard and the cut-off value was set at 60 mL/min/1.73 m^2^ for CKD as defined by the National Kidney Foundation [6]. To compare the creatinine-based and combined estimations of the GFR (C&G, MDRD, CKD-EPI, and CKD-EPI creatinine and cystatin formulas) with ^51^CrEDTA clearance and the serum cystatin C-based estimation the Bland and Altman plots were used [32]. The mean difference between estimated and measured GFR values estimates the global bias. The width of SD of the mean difference is an estimation of precision. The accuracy within 30% for different equations was measured as the percentage of results that did not deviate more than 30% from the measured GFR with ^51^CrEDTA clearance. The accuracy within 30% of stages of CKD was analyzed. The ability to correctly estimate the patient's GFR below and above 60 mL/min/1.73 m^2^ with different equations compared to the “gold standard” was also analyzed.

The study protocol was in conformity with ethical guidelines and informed consent was obtained from each participant.

## 3. Results

**Figure 1:**
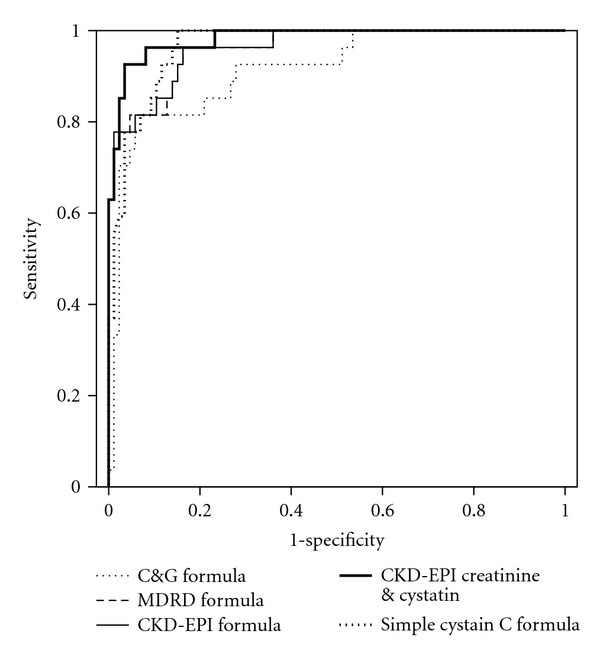
ROC curve analysis of diagnostic accuracy of calculated clearance from the C&G formula, the MDRD formula, the CKD-EPI formula, the CKD-EPI creatinine and cystatin formula, and the simple cystatin C formula. The GFR determined with ^51^CrEDTA was used as the gold standard and cut-off value was set at 60 mL/min/1.73 m^2^.

**Figure 2:**
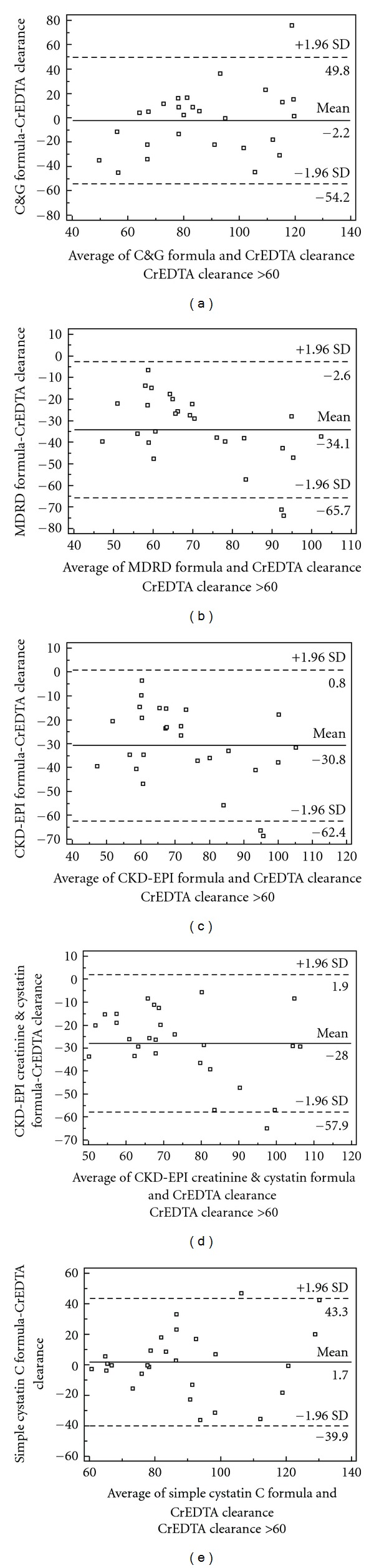
Bland and Altman plot for differences between estimated GFR and measured GFR. On the *x*-axis, the average GFR is given and on the *y*-axis the difference in mL/min/1.73 m^2^ between the estimated GFR, derived from (a) C&G and (b) MDRD formula, (c) CKD-EPI formula, (d) CKD-EPI creatinine and cystatin formula, (e) simple cystatin C formula is given. The mean difference & the 1.96 SD limits are plotted.

The age of patients ranged from 27 to 86 years, giving a mean age of 64 ± 10.2 years. Their heights ranged from 149 to 189 cm and the mean height was 167 ± 9 cm. The mean weight was 87.5 ± 15 kg, with patients ranging from 59 to 142 kg. The mean body mass index of patients was 31.3 ± 4.8 kg/m^2^ (women 31.6 ± 4.8 kg/m^2^; men 31 ± 4.8 kg/m^2^). The mean ^51^CrEDTA clearance in our patients was 42.9 ± 30 mL/min/1.73 m^2^, ranged from 5 to 130 mL/min/1.73 m^2^. Two-thirds (76%) of enrolled patients have ^51^CrEDTA clearance under 60 mL/min/1.73 m^2^. The mean serum creatinine concentration value was 230.6 *μ*mol/L, and ranged from 66 to 674 *μ*mol/L (SD ± 127.6). The serum cystatin C concentration values were between 0.66 and 6.99 mg/L; the mean value was 2.47 mg/L (SD ± 1.25). Statistically significant correlation was found between ^51^CrEDTA clearance and GFR calculated from the C&G formula (*r* = 0.792; *P* < 0.0001), the MDRD formula (*r* = 0.897; *P* < 0.0001), the CKD-EPI formula (*r* = 0.899; *P* < 0.0001), the CKD-EPI creatinine and cystatin formula (*r* = 0.924; *P* < 0.0001), and the simple cystatin C formula (*r* = 0.877; *P* < 0.0001). In a comparison of the correlation coefficients we found that the correlations between ^51^CrEDTA clearance and the simple cystatin C formula and ^51^CrEDTA clearance and the MDRD formula or the CKD-EPI formula and ^51^CrEDTA clearance were superior to the correlation between ^51^CrEDTA clearance and the C&G formula (*P* = 0.034 for the simple cystatin C formula, *P* = 0.0048 for the CKD-EPI formula, *P* = 0.0001 for the CKD-EPI creatinine and cystatin formula and *P* = 0.0038 for the MDRD formula). No difference between correlation coefficients of the MDRD, CKD-EPI, or CKD-EPI creatinine and cystatin formulas and the simple cystatin C formula was found (*P* = 0.4856, *P* = 0.4390, *P* = 0.06). Diagnostic accuracy (area under the ROC curves, sensitivity, and specificity) at the cut-off value for GFR 60 mL/min/1.73 m^2^ of the different creatinine-based equations, combined equation, and the simple cystatin C-based equation are presented in Table 1. The ROC curve analysis (cut-off for GFR 60 mL/min/1.73 m^2^) showed that the simple cystatin C, MDRD, CKD-EPI, CKD-EPI creatinine and cystatin formulas had bigger area under the curve than the C&G formula, but no statistically significant differences between the formulas was found (Table 1, Figure 1). Bland and Altman analysis for the same cut-off value showed that creatinine formulas (the C&G formula bias: −2.2 mL/min/1.73 m^2^; the MDRD formula bias: −34.1 mL/min/1.73 m^2^; the CKD-EPI formula: −30.8 mL/min/1.73 ^2^; the CKD-EPI creatinine, and cystatin formula: −28 mL/min/1.73 m^2^) underestimated and the simple cystatin C formula (bias: 1.7 mL/min/1.73 m^2^) slightly overestimated measured GFR. Analysis of the SD of the mean difference between the estimated and measured GFR showed that all equations lacked precision. It was 26.5, 16.1, 16.1, and 15.3 mL/min/1.73 m^2^ for the C&G, MDRD, CKD-EPI, and CKD-EPI creatinine and cystatin formulas, and 21.2 mL/min/1.73 m^2^ for the simple cystatin C formula (Table 2). The accuracy within 30% of estimated ^51^CrEDTA clearance values differs according to stages of CKD (Table 3, Figure 2). In patients with stages 1 and 2 of CKD higher accuracy within 30% was found for the simple cystatin C formula compared to all serum creatinine-based formulas and combined formula, but the differences were not statistically significant. Contrary, in patients with moderate impaired kidney function and stages 4 and 5 of CKD higher accuracy within 30% was found for the serum creatinine-based formulas and combined formula compared to accuracy for the simple cystatin C formula.

Analysis of ability to correctly predict patient's GFR below or above 60 mL/min/1.73 m^2^ showed the higher ability for the simple cystatin C formula compared to the C&G, MDRD formulas but the differences were not statistically significant (Table 2).

## 4. Discussion

The current Kidney Disease Outcomes Quality Initiative (K/DOQI) guidelines emphasize the need to assess kidney function using the predictive creatinine-based equations rather than just serum creatinine [6]. The C&G and MDRD formulas have been evaluated in numerous previously published studies and widely applied. However, the formulas have some well-known limitations [3]. Therefore, new alternatives like creatinine-based CKD-EPI equation, cystatin C-based formulas, and equation that use both serum creatinine and serum cystatin C were developed [5, 18, 19, 22–26, 31, 33, 34]. In our study we compared the all three, widely used creatinine-based equation, equation that use both serum creatinine and serum cystatin C, and one very simple cystatin C-based equation in well-defined, overweight patients with DM2 and CKD. Some previously published studies suggest that body weight explain some of the difference in the ability of serum creatinine-based equations for estimation of GFR in overweight population with DM2 [27, 28]. Furthermore, it is known that in obese patients with DM2 C&G formula clearly overestimated measured GFR [35, 36]. Proposed explanation for this is that C&G formula estimates GFR as proportional to body weight; omitting weight from the calculation improved its correlation to GFR and therefore increased the bias [37, 38]. However, less is known about the ability of newer equations, like CKD-EPI formulas and serum cystatin C-based equations, to estimate the GFR in overweight patients with DM2. We have shown that the simple cystatin C formula achieved at least as good diagnostic performance as the creatinine-based formulas, including newer CKD-EPI formulas. Some others studies on population with DM2 showed a higher accuracy of the cystatin C formulas compared to the C&G and MDRD formulas [20, 21, 24, 31]. Some authors even concluded that the cystatin C formula is complementary to the serum creatinine-based equations or can be used in place of the serum creatinine-based equations [21]. Stevens et al. showed that the CKD-EPI creatinine-based equation is more accurate than MDRD study equation across various study populations and clinical conditions, but no such data is available for the CKD-EPI formulas compared to serum cystatin C-based equations in well-defined population DM2 patients [9, 26]. In our present study in population of overweight DM2 patients with impaired kidney function the correlation between the “gold standard”, ^51^CrEDTA clearance and the simple cystatin C formula was better than the correlation between the ^51^CrEDTA clearance, and GFR calculated with the C&G formula. No difference between correlation coefficients of the MDRD formula or the CKD-EPI formulas and the simple cystatin C formula was found. According to our results, the simple cystatin C formula had large area under curve for cut-off value GFR 60 mL/min/1.73 m^2^ but no statistically significant difference in diagnostic accuracy between the simple cystatin C formula and creatinine-based formulas or CKD-EPI formulas was found. The Bland and Altman analysis for same cut-off value showed that all three creatinine-based formulas and CKD-EPI creatinine and cystatin formula underestimated the measured GFR (^51^CrEDTA clearance). On the contrary, the simple cystatin C formula overestimated measured GFR for only 1.7 mL/min/1.73 m^2^. The accuracy within 30% of the estimated gold standard values demonstrated the superiority of the simple cystatin C formula compared to the MDRD and CKD-EPI formulas only in patients with mild impaired kidney function. Furthermore, in the analysis of the ability to correctly predict patient's GFR below or above 60 mL/min/1.73 m^2^ the higher ability for the simple cystatin C formula compared to the C&G, MDRD formulas was found but the differences were not statistically significant. Finally, the higher ability to correctly predict patient's GFR for CKD-EPI formulas compared to the simple cystatin C was found but again the differences were not statistically significant.

The results of the present study suggest that the cystatin C-based prediction equation, which requires just one variable (serum cystatin C concentration), achieved a diagnostic performance that was at least as good as the creatinine-based formulas using more variables. In our overweight DM2 patients with CKD, the simple cystatin C formula overrode well-known tendency of creatinine-based formulas and combined formula to underestimate patient's GFR, what can lead to unnecessary diagnostic and therapeutic strategies regarding to the stage of CKD. The newest sophisticated CKD-EPI formulas like C&G and MDRD formulas request additional calculator equipment which is absolutely needless by using the simple cystatin C formula.

Our study has some potential limitations. First, result of present study analyzes Caucasian population only. The creatinine-based equations and combined formula were developed from studies which involved participants of all races. Thus, direct comparison of equations can be performed between CKD-EPI formulas (equations include variable on the basis of race) and the simple cystatin C formula only. Second, mentioned studies were performed with different GFR references and gold standards. Some authors used ^125^I-iothalamate (Levey et al. [9], Hoek et al. [18], Perkins et al. [31]); others used Iohexol (Grubb et al. [17], Tidman et al. [23]) as the “gold standard” for the measuring of the GFR. In our study, like in the study published by Chudleigh et al. [24], ^51^CrEDTA clearance was used for estimation of GFR. Therefore, an exact direct comparison between these studies is difficult. Third, in our study the serum cystatin C and the serum creatinine were measured only once and so we cannot rule out known intrapatient variability of serum creatinine or serum cystatin C concentration, which can be present due to production and/or extra renal elimination. Fourth, in our study, a nephelometric assay was used to measure serum cystatin C concentration, while some other investigators have used colorimetric or turbidimetric assays. However, the standardization of serum cystatin C measurements is solved now and internationally accepted reference material is available [39–41]. Finally, the cause of kidney damage other than diabetes mellitus was not analyzed in our patients.

In conclusion, our study demonstrated that the simple cystatin C formula could be useful tool for evaluation of renal function in overweight patients with DM2 and impaired kidney function in daily clinical practice in hospital and especially in outpatients. Despite the advantages of the simple cystatin C formula, cystatin C-based equations cannot completely replace the “gold standard” for estimation of the GFR in a population of DM2 patients with CKD, but may contribute to a more accurate selection of patients requiring such invasive and costly procedures.

## Figures and Tables

**Table 1:** Diagnostic accuracy (area under the ROC curves, sensitivity, specificity) and comparison of ROC curves at cut-off value for GFR 60 mL/min/1.73 m^2 ^of calculated clearance from the C&G formula, the MDRD formula, the CKD-EPI formula, the CKD-EPI creatinine and cystatin formula, and the simple cystatin C formula. The GFR determined with ^51^CrEDTA was used as the gold standard.

Equation	AUC	Sensitivity %	Specificity %	P* value
C&G formula	0.915	93.0	81.5	0.162
MDRD formula	0.961	96.3	83.7	0.866
CKD-EPI formula	0.962	83.7	96.3	0.891
CKD-EPI creatinine and cystatin formula	0.982	92.6	96.5	0.322
Simple cystatin C formula	0.966	84.9	100	

P* calculated according to the simple cystatin C formula.

AUC: area under the curve.

**Table 2:** Bias, precision (cut-off value for GFR 60 mL/min/1.73 m^2^), and ability to correctly predict patient's GFR according to ^51^CrEDTA clearance in 113 patients with diabetes mellitus type 2 and chronic kidney disease.

Equation	Bias (mL/min/1.73 m^2^)	Precision (mL/min/1.73 m^2^)	Ability to correctly predict patient's GFR below or above 60 mL/min/1.73 m^2^
C&G formula	−2.2	26.5	83.2%
MDRD formula	−34.1	16.1	86.7%
CKD-EPI formula	−30.8	16.1	92.9%
CKD-EPI creatinine and cystatin formula	−28	15.3	89.4%
Simple cystatin C formula	1.7	21.2	88.5%

**Table 3:** The accuracy of formulas within 30% of estimated ^51^CrEDTA clearance values for different stages of CKD.

	Stages of chronic kidney disease				
CKD stage (number of patients) GFR, mL/min/1.73 m^2^	Stage 1 (10) ≥90	Stage 2 (17) 60–89	Stage 3 (36) 30–59	Stage 4 (39) 15–29	Stage 5 (11) <15
Equation	Accuracy within 30% of estimated ^51^CrEDTA clearance (%)				

C&G formula	90.0	70.6	55.6	43.6	27.3
MDRD formula	20.0	58.8	69.4	69.2	63.6
CKD-EPI formula	20.0	58.8	69.4	66.7	54.5
CKD-EPI creatinine and cystatin formula	30.0	52.9	63.9	71.8	72.7
Simple cystatin C formula	80.0	82.4	63.9	23.1	0.0
